# Construction of a BALB/c-Nu Mouse Model of Invasive Bladder Carcinoma and Preliminary Studies on the Treatment of Bladder Tumors through Internal Iliac Arterial Infusion of Albumin-Bound Arsenic Trioxide (As_2_O_3_)

**DOI:** 10.1371/journal.pone.0124959

**Published:** 2015-04-27

**Authors:** Yunlong Li, Guopeng Yu, Qiaoxing Li, Weilu Wang, Xiangqian Shen, Hua Liu, Ruijiang Liu

**Affiliations:** 1 Department of Urology, Affiliated Kunshan Hospital of Jiangsu University, Kunshan, Suzhou 215300, P.R. China; 2 Department of Urology, Huashan Hospital, Fudan University, Shanghai 200040, P.R. China; 3 Fudan Institute of Urology, Fudan University, Shanghai 200040, P.R. China; 4 Department of Neurosurgery, Affiliated Kunshan Hospital of Jiangsu University, Kunshan, Suzhou 215300, P.R. China; 5 School of Material Science and Engineering, Jiangsu University, Zhenjiang 212013, P.R. China; National Cheng Kung University, TAIWAN

## Abstract

To establish a BALB/c-nu mouse model of invasive bladder carcinoma and to investigate the feasibility, efficacy, and side effects of treating the mouse xenografts with internal iliac arterial infusion of albumin-bound arsenic trioxide (As_2_O_3_). Bladder tumors were established by intravesicular injection. Color Doppler were used to monitor tumor growth. Albumin-bound As_2_O_3_ and bovine serum albumin (BSA) nanoparticles were synthesized by cross-linking. BALB/c-nu mice were randomly divided into four treatment groups: 1) normal saline, 2) BSA nanoparticles, 3) As_2_O_3_ injections, and 4) albumin-bound As_2_O_3_. In an attempt to replicate the treatment of bladder cancer in humans using internal iliac arterial infusion, the drugs were injected into the mouse abdominal aorta. Tumor xenografts were established successfully. Mice treated with As_2_O_3_ injections and with albumin-bound As_2_O_3_ had significantly smaller bladders (36.59% and 37.82% smaller, respectively) than mice given normal saline injections (P < 0.01). Mice receiving As_2_O_3_ injections had lower white blood cell (WBC) and platelet counts compared with mice receiving normal saline injections only (P < 0.05). However, mice treated with albumin-bound As_2_O_3_ did not experience a significant decrease in WBC or platelet counts compared with control mice. A model of intra-arterial bladder cancer treatment was successfully established in BALB/c-nu mice. In this model, albumin-bound As_2_O_3_ appeared to be an effective method for treating bladder tumors, with less severe hematologic side effects compared with As_2_O_3_ alone. The infusion of albumin-bound As_2_O_3_ through the internal iliac artery is a promising method of bladder cancer therapy.

## Introduction

Advanced-stage bladder cancer is difficult if not impossible to cure with surgery alone and frequently develops resistance to chemotherapy. More effective chemotherapy is clearly needed to improve cure rates in this disease. Arsenic trioxide (As_2_O_3_) is commonly used for the treatment of acute promyelocytic leukemia (APL)[1–3]and has recently been investigated as an agent for the treatment of multiple solid tumors[4–8].As_2_O_3_ appears to have activity against bladder cancer cells in vitro and as an intravesicular injection for the treatment of superficial bladder tumors. However, intravenous injections of As_2_O_3_ at therapeutic concentrations cause serious adverse reactions. To decrease the toxicity of treatment and increase the therapeutic effect, we prepared albumin-bound As_2_O_3_ and injected the drug through the abdominal aorta in order to imitate the optimum method of administration in the clinic. This study combines traditional Chinese and Western medical techniques for the development of a promising new technique for the treatment of bladder cancer.

## Material and Methods

### Cell line and laboratory animals

The human bladder cancer cell line EJ, obtained from Nanjing KeyGen Biotech Co., was cultured in a RPMI 1640 medium with 10% fetal bovine serum (Sigma Chemicals). Cell concentration was adjusted to 1×10^6^/ml of living cells once the cells were in logarithmic growth phase. A hypodermic injection was performed. Tumor injections were derived from the cell suspension under sterile conditions, and the cell concentration for injection was adjusted to 1×10^7^/ml (in PBS). Four- to six-week-old female BALB/c-nu mice, weighing approximately 16–20 g, were provided by the experimental animal center of Guangxi Medical University. All experiments were performed in accordance with the institution’s animal ethics guidelines. (Certificate of quality No: SCXK (GUI)2009-0002.) Animal use and care protocols, including all operation procedures, were approved by the Animal Care and Use Committee of Soochow University and conformed to the *Guide for the Care and Use of Laboratory Animals* issued by the National Institutes of Health. The protocols were also conducted in accordance with the *Guidance Suggestions for the Care and Use of Laboratory Animals*, formulated by the Ministry of Science and Technology of China[9].

### Main instruments and reagents

The main instruments and reagents that were used in the study included the following: 3K15 freezing centrifuge (Sigma Chemicals); NANOPHOX(0150 P) Laser Diffraction Size Analyzer (SYMPATEC GmbH); Philips Achieva 3.0T X-series MRI; GE LOGIQ9 color Doppler; LH 750 automated hematology analyzer and LX20 automatic chemistry analyzer (Beckman Coulter Inc.); Highly enriched A_2_O_3_ (Sigma Chemicals); Arsenic trioxide injection (Beijing SL Pharmaceutical Co. Ltd.); Iopromide Injection (Schering Pharmaceutical Limited); other domestic analytical reagents.

### Establishment of the animal model

**Fig 1:**
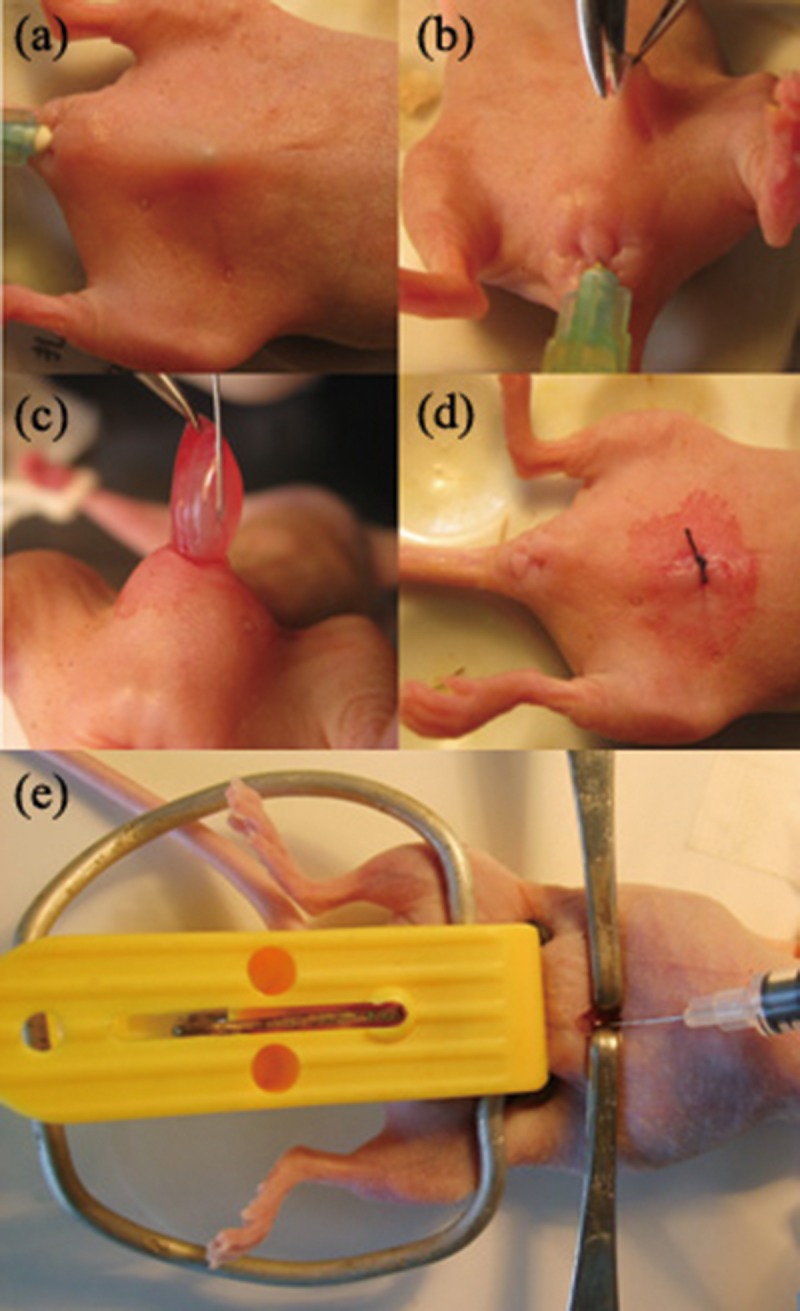
Establishment and treatment methods of BALB/c-nu mouse model of invasive bladder carcinoma. (a) Insert the hard elbow urinary catheter into the bladder to determine the surgical incision. (b) Cut the abdominal wall layer by layer. (c) Inject tumor cells into the bladder wall. (d) Suture the incision with 4–0 nylon. (e) Inject the drugs into the abdominal aorta of the BALB/c-nu mouse by using a 1 mL insulin syringe.

In attempt to improve the method of Li et al.[10], a hard elbow urinary catheter was designed by the investigators to simplify the injection of tumor cells into the mouse bladders. We established a BALB/c-nu mouse model of invasive bladder carcinoma by using minimal invasive techniques that we drew 20μl (1×10^7^/ml) tumor cell suspension with microinjector and injected into the bladder wall (Fig 1).

### Preparation of albumin-bound As_2_O_3_

**Fig 2:**
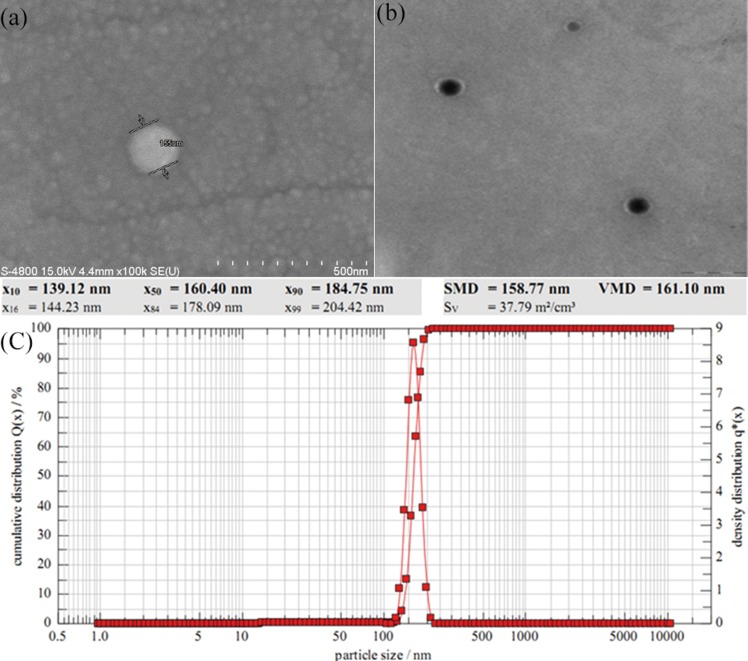
Albumin-bound As_2_O_3_ microscopy images and Laser Diffraction Size Analyzer. (a), (b) Show albumin-bound As_2_O_**3**_ microscopy images by scanning electron microscope and transmission electron microscope respectively. (c) Laser Diffraction Size Analyzer showed the particle size of albumin-bound As_2_O_**3**_.

According to the method of Zhou et al.[11], the oily and water phases were prepared. After phacoemulsification for 15 minutes on 400 watt power, a high speed homogenizer was used to stir the solution for 3 minutes at a velocity of 23000 rpm. Immediately after this, the homogenate was placed into 2 mL glutaraldehyde and toluene saturated solution to stir and solidify for 6 hours at low speed (about 500 rpm). After this step, the mixture was centrifuged at 500 rpm and 1000 rpm for 5 minutes, allowing the larger microspheres to be removed. The mixture was centrifuged again at 5500 rpm for 5 minutes. Following this step, cyclohexane and acetone were used to wash the solution 3 times. Microspheres were then collected and subjected to dry heat sterilization for 4 hours at 120°C[12].The microspheres were then re-suspended in saline. Uniform dispersion of the microspheres was verified by microscopy at high magnification. Particle disparity was mostly in the range of 180 nm (Fig 2), and the drug loading range was 6.55%. BSA nanoparticles were prepared in a similar fashion.

### Experimental classification

Fifty female BALB/c-nu mice, approximately week old and body weight, were chosen at random to establish the animal model. Tumor growth was measured with color Doppler and MRI on the tenth day after the EJ cell transplantation procedure. Forty female BALB/c-nu mice with similar tumor sizes were chosen to be randomly divided into four equal groups for intra-arterial injection of normal saline, BSA nanoparticles, As_2_O_3_ injection, or albumin-bound As_2_O_3_.

### Internal iliac arterial infusion of albumin-bound As_2_O_3_

On the eleventh day after tumor injection, the mice were anesthetized, and the operative sites were sterilized as detailed above. A surgical incision was made to open the abdominal cavity, and a mouse abdominal wall retractor (designed by the investigator) was used to retract the omentum and intestines into the right upper quadrant of the abdominal cavity, exposing the abdominal aorta. The aorta was occluded, and at the same time, a vascular clip device (also made by the investigator) was used to compress both external iliac artery branches in order to prevent the flow of drug into these vessels. Drugs were injected into the abdominal aorta using a 1 mL insulin syringe (Fig 1E). After finishing the injection, the needle was withdrawn, and the entry point was compressed for 1 minute. Once bleeding had stopped, the arterial clips were removed. Then, 1mL of low molecular weight dextran was injected into the abdomen to prevent adhesions. The incision was closed with layers of interrupted sutures. Upper abdominal incision with drug injection was performed at day 3 after the operation, and at day 6 after the operation, injections were performed via a lateral rectus abdominis muscle incision. On day 9, the cycle of injections was repeated beginning with the original midline incision. A total of six injections were performed. After the operation, all the mice were maintained in a laminar air-flow cabinet under pathogen-free conditions.

### Color Doppler

**Fig 3:**
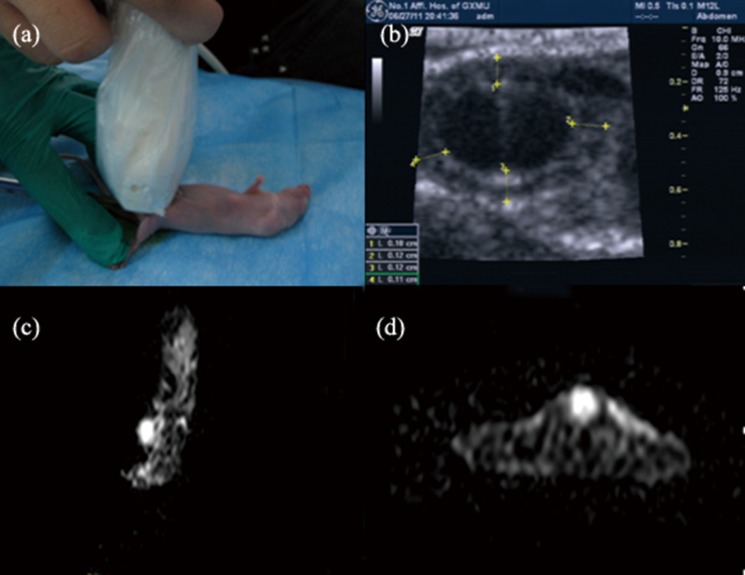
Color Doppler and MRI detection of bladder cancer. (a) The color Doppler ultra-wide-range linear-array transducer with frequency 12 MHz was used to scan the bladder of BALB/c-nu mice. (b) Color Doppler showed bladder filled well and thickening of the bladder wall. (c, d) MRI detected thickening of the bladder wall and various degrees of bladder filling defects.

The color Doppler ultra-wide-range linear-array transducer with frequency 12 MHz was then used to scan the bladder (Fig 3A).

### MRI examination

MRI of the bladder was performed according to scanning parameters that have been described previously[13].

### Observation time

Diet and mental state were observed every day. Bedding was changed, and dressing changes were performed on alternate days. Twenty-eight days after the initial implant of bladder cancer cells, the experiment was concluded, and all the BALB/c-nu mice were sacrificed.

### Biochemical analysis of the BALB/c-nu mice

At the end of experiment, blood samples were collected from the hearts of all the mice. Routine blood counts and chemistries were performed, including white blood cell count (WBC), hemoglobin (Hb) levels, platelet (PLT) count, alanine aminotransferase (ALT), serum total protein (TP), and blood urea nitrogen (BUN) levels.

### Pathological examination

The urethra was ligatured after 10% formaldehyde solution was infused into the bladder per the urethra. The bladder was excised and cut into 4–5 μm sections after fixation in paraffin and routine H&E staining. Pathologic examinations of the lungs, liver, kidney, spleen and pelvic lymph nodes were also performed.

### Tumor growth inhibiting rate and relative weight determination of bladder

Inhibitory rate was calculated using the following formula:

[(bladder weight in the normalsalinegroup-bladder weight in thetreatmentgroup)/bladder weight in the normalsalinegroup]×100%.

Bladder relative weight was calculated using the following formula:

bladderweight/total bodyweight.

### Statistical Analysis

Measurement data were measured by (X±s). Statistical analysis was performed using SPSS 13.0 software. A *p* value of less than 0.05 was considered to be statistically significant.

## Results

### General conditions

Following drug injection, one mouse in each treatment group died of excessive anesthesia, and one mouse in each group died of infection. Twenty-six days after tumor cell injection, all surviving mice in the normal saline-treated control group were obviously thin, with low spirits and rigid back arching. Three of the mice were on the edge of death. Mice in the BSA nanoparticle-treated control group were in similar condition, with two mice on the edge of death. Mice receiving As_2_O_3_ injection (7) had less severe symptoms than the control mice. Mice in the albumin-bound As_2_O_3_ group (8) were in good spirits, and their eating, drinking and relieving were normal.

### Results of MRI and color Doppler

On the tenth day after EJ cell transplantation, MRI detected thickening of the bladder wall and various degrees of bladder filling defects (Fig 3C and 3D). Color Doppler showed similar results, with significant thickening of the bladder wall (Fig 3C).

### Pathohistological changes

**Fig 4:**
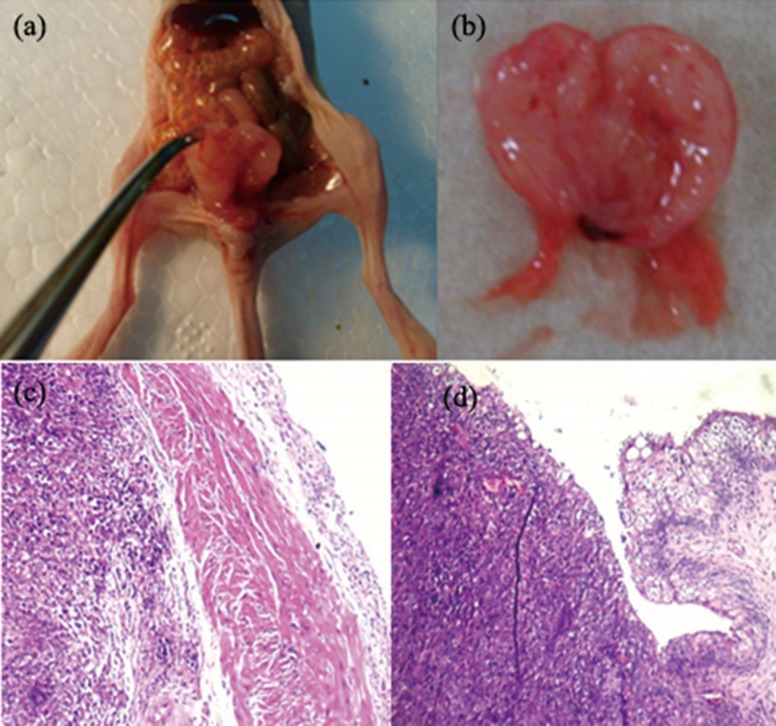
Pathological examination. (a), (b) Gross photographs show the mouse bladder was invaded by the tumor cells and enlarged. And bladder wall was diffuse thickening. (c) Pathological examination showed tumor with extensive serous membrane, muscularis propria diffuse invasion, tumor cells with loss of polarity and arranged disorder. This showed significant cytologic atypia whithout obvious necrosis. And it was solid tumor on the left side (H×200). (d) Tumor cells invading the submucosa and muscular layer on the left side. The enlarged nuclei of tumor cells showed pleomorphism with hyperchromatic, irregular nuclei, inconsistency of shape and size, irregular clumped chromatin with uneven distribution, increased ratio of nucleus to cytoplasm, and prominent enlarged nucleoli. And it was normal mucosa on the right side (H×400).

At the end of experiment, mice had developed thickened bladder walls and had solid masses within the bladder (Fig 4A and 4B). Pathological examination showed tumor had extensive serous membrane and muscularis propria diffuse invasion, with tumor cells loss of polarity and arranged disorder. This showed significant cytologic atypia. The enlarged nuclei of tumor cells showed pleomorphism with hyperchromatic, irregular nuclei, inconsistency of shape and size, irregular clumped chromatin with uneven distribution, increased ratio of nucleus to cytoplasm, and prominent enlarged nucleoli.(Fig 4C and 4D). No metastases were found in the lungs, liver, kidney, spleen or pelvic lymph nodes.

### Growth inhibition of bladder tumor

Because the tumors were obviously invading normal structures in most of the mice, the whole bladders were sectioned for comparison. The average bladder weight in mice receiving As_2_O_3_ injections was 41.68±1.72 mg and in mice treated with albumin-bound As_2_O_3_ was 40.87±1.90 mg. These weights are significantly lower than the bladder weights in mice receiving normal saline injections (P < 0.01). There was no marked difference between mice treated with normal saline and those receiving BSA nanoparticles. The tumor inhibitory rates were 36.59% and 37.82% in mice receiving As_2_O_3_ and those receiving albumin-bound As_2_O_3_, respectively (Table 1).

**Table 1:** Bladder weights and inhibitory rates observed in 4 BALB/c-nu mice (%).

Group	Bladder absolute weight (mg)	Bladder relative weights (mg/g)	Inhibitory rates (%)
Normal saline	65.73±2.71	3.65±0.16	
BSA nanoparticles	64.98±2.78	3.68±0.29	
As_2_O_3_ injection	41.68±1.72**	2.36±0.13**	36.59
Albumin-bound As_2_O_3_	40.87±1.90**	2.27±0.13**	37.82

(Compared with the normal saline group: **P<0.01)

### Side effects (hematologic and blood chemistries)

Mice who received As_2_O_3_ injections and those which received albumin-bound As_2_O_3_ had lower WBC, platelet counts, and hemoglobin levels compared with mice receiving normal saline injections (Table 2). Compared with the albumin-bound As_2_O_3_ group, mice in the As_2_O_3_ injection had lower WBC (P < 0.05) and platelet counts (P < 0.01). Mice receiving As_2_O_3_ injection had elevated ALT compared with the normal saline,but albumin-bound As_2_O_3_ groups was the highest among the four groups. Based on an analysis to the Data of ALT, it is concluded from the statistical analysis that the four groups were not significant difference each other (P>0.05). Total protein and BUN levels were similar among all treatment groups.

**Table 2:** Effects of different treatments on blood counts and routine biochemical parameters.

Group	WBC(×109/L)	PLT(×109/L)	Hb(g/L)	ALT(U/L)	TP(g/L)	BUN(mmol/L)
Normal saline	5.05±0.22	421.19±9.62	124.81±2.44	72.56±3.36	48.26±4.09	7.53±0.39
BSA nanoparticles	5.00±0.25	416.91±9.46	124.61±2.71	71.75±4.68	46.21±6.27	7.35±0.35
As_2_O_3_ injection	2.45±0.39**	330.80±14.41**	120.70±4.01*	73.29±3.40	45.13±3.32	7.56±0.23
Albumin-bound As_2_O_3_	4.56±0.28**^△△^	408.15±13.43*^△△^	121.55±4.26	75.25±3.85	45.03±3.25	7.18±0.30

(Compared to the normal saline group: **P<0.01;*P<0.05)

(Compared to the As_2_O_3_ injection and albumin-bound As_2_O_3_ groups: △△P<0.01)

## Discussion

Effective translational research is essential in bringing promising in vitro cancer therapies into the clinic[14]. This study attempted to use an animal model to test a new formulation of As_2_O_3_ (albumin-bound), administered via an existing clinical treatment method (intra-arterial chemotherapy injection). This study design allowed us to evaluate the cytotoxicity of this therapy, observe side effects, and test the efficacy of this particular animal model.

Invasive bladder cancer refers to cancers classified as T_2_, T_3_, and T_4_ stages, representing up to 20% of bladder cancers[15, 16].The American cancer association shows that there were around 70530 new bladder cancer patients, and 14680 patients died of bladder cancer in 2010[17].In the all-male tumors, the incidence of bladder cancer was the fifth, but it was the eighth in the women tumors[18]. Radical resection is the major treatment for invasive bladder cancer, but surgical mortality may be as high as 34%[19].Bladder cancer patients tend to be older, with multiple medical comorbidities, and many are not able to tolerate surgical resections[20].Research is increasingly focused on the development of new chemotherapy drugs and techniques of administration[21].Considering this goal, we performed an animal study by establishing a BALB/c-nu mouse model of invasive bladder carcinoma and treating it through internal iliac arterial infusion of albumin-bound As_2_O_3_. A major advantage of this technique is the fact that it allows the achievement of a high drug concentration in the lymphatic vessels and microcirculation of the tumor[22].

**Fig 5:**
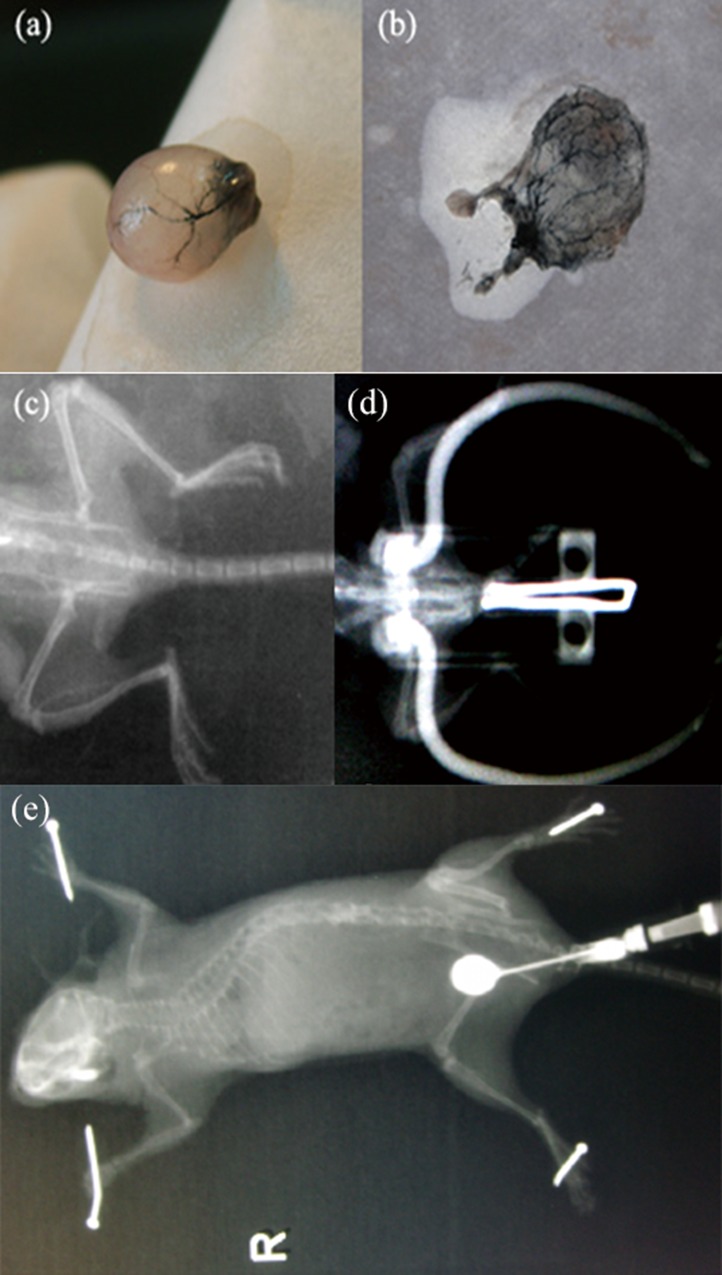
Simulation of drug pathway by internal iliac arterial infusion. (a, b) Ink was injected into aorta abdominalis of the BALB/c-nu mice and the bladder artery was clearly. (c) After cutting the bladder, the main branches filled ink was clearly manifested within the artery of bladder mucous membrane. (d) The BALB/c-nu mice pelvic X-ray plain film was showed and it was lower density in pelvic region. (e) Imaging showed that contrast was concentrated in the pelvic blood vessels and bladder region after a contrast medium of iopromide was injected.

Originally, we attempted to inject the drugs through the femoral artery. However, the diameter of the mouse femoral artery is smaller than the needle of a 1mL syringe, making this impossible. Therefore, we injected through the abdominal aorta under a microscope while occluding the bilateral external iliac arteries to prevent bypass circulation of the drug. For the preliminary experiments, x-rays were taken immediately after a contrast medium of iopromide was injected using the above technique. Imaging showed that contrast was concentrated in the pelvic blood vessels and bladder region (Fig 5D). Similarly, the bladder artery was clearly visible after injecting ink (Fig 5A and 5B). As_2_O_3_ is the major active ingredient of arsenic, an important element of traditional Chinese medicine. Arsenic has been considered a highly poisonous drug and is not widely utilized in Western medicine[23]. However, because As_2_O_3_ is effective in the treatment of acute promyelocytic leukemia (APL), it has become a subject of increasing oncologic investigation. It is the main cause that unlimited proliferation[24]. As_2_O_3_ can induce tumor cells apoptosis[25].Arsenic may inhibit the growth, invasion and metastasis of tumor but have minimal effects on normal cells, and it has been shown to have a powerful antitumor effect without cross-resistance between other chemotherapeutics[26].Arsenic’s effect on apoptosis correlates positively with drug concentration and exposure time[27–29].

In this new dosage form, preparation of albumin-bound As_2_O_3_ was completed by cross-linkage. Ahn, et al[30] used liposomes vesicles making nanoparticles to encapsulate As_2_O_3_, which could improve As_2_O_3_ pharmacokinetics in vivo and anti-tumor effect, and provides the basis for the clinical application. The albumin nanoparticles drug delivery system is the most compelling in the field of antitumor drug, which can increased the targeted, improve curative effect, reduce side effects[31].We adjusted the parameters of dispersion speed, rate of lipid/aqueous phases, concentration of albumin and agitation/solidification times. Previous reports have described the filtration of nanoballs with a 0.22 μm membrane. However, large microspheres may block these filters, rendering them ineffective. In this report, we describe a different technique for creating nanoballs of uniform diameter.

In this experiment, we demonstrated significant shrinkage of bladder tumors in mice through internal iliac arterial infusion of albumin-bound As_2_O_3_ with minimal adverse effects. As_2_O_3_ injections had lower white blood cell (WBC) and platelet counts compared with mice receiving normal saline injections only, which caused serious myelosuppression. The ALT, TP, and BUN levels were not significantly different between the groups treated with albumin-bound As_2_O_3_ and As_2_O_3_ injections. In the case of active targeting, the albumin contained in the nanoparticle albumin-bound (nab)-paclitaxel is thought to facilitate receptor-mediated endothelial transcytosis of albumin-bound plasma constituents or albumin-based nanoparticles into the extravascular space. Eventually, the albumin will be actively and selectively recognized and bound by the proteins-rich tumor tissues.

When the albumin-bound As_2_O_3_ flows into the circulatory system, it is released slowly and the suppressive effect of the bone marrow cells can be reduced considerably. The entry of injected As_2_O_3_ into the blood vessels of the bladder results in instantaneous very high increase in the concentration of As_2_O_3_ around the bladder cancer cells; this high concentration of As_2_O_3_ destroys the tumor cells. The As_2_O_3_ concentration drops gradually as it enters the bloodstream. Approximately 95%of the drug integrates with hemoglobin and is distributed to all parts of the body, leading to a decrease in WBC and PLT counts and in Hb levels. In this experiment, the BALB/c-nu mouse model for invasive bladder carcinoma implanted with human bladder cancer cells was treated with infusion of albumin-bound As_2_O_3_ through the internal iliac artery. In the human body, the internal iliac artery has a relatively slow blood flow and it allows the direct delivery of chemotherapeutic agents into the bladder[26], making it the preferred vessel for delivering antineoplastic drugs into the bladder[32].

## Conclusion

This experiment demonstrates the successful establishment of a BALB/c-nu mouse model of invasive bladder carcinoma, and shows that albumin-bound As_2_O_3_ is a promising drug for the treatment of bladder cancer. Further study of intra-arterial injection of albumin-bound As_2_O_3_ is needed before this therapy can be utilized in humans. However, we are hopeful that this promising therapy can be brought to the clinic.
